# Person-Specific Analyses of Smartphone Use and Mental Health: Intensive Longitudinal Study

**DOI:** 10.2196/59875

**Published:** 2025-02-26

**Authors:** Merve Cerit, Angela Y Lee, Jeffrey Hancock, Adam Miner, Mu-Jung Cho, Daniel Muise, Anna-Angelina Garròn Torres, Nick Haber, Nilam Ram, Thomas N Robinson, Byron Reeves

**Affiliations:** 1 Graduate School of Education Stanford University Stanford, CA United States; 2 Department of Communication Stanford University Stanford, CA United States; 3 Cyber Policy Center Stanford University Stanford, CA United States; 4 Department of Psychiatry and Behavioral Science Stanford University Stanford, CA United States; 5 Center for Survey Research Research Center for Humanities and Social Sciences Academia Sinica Taipei Taiwan; 6 Screenlake Inc San Mateo, CA United States; 7 Department of Computer Science Stanford University Stanford, CA United States; 8 Department of Psychology Stanford University Stanford, CA United States; 9 Departments of Pediatrics Medicine and Epidemiology & Population Health Stanford University Stanford, CA United States

**Keywords:** media use, mental health, mHealth, uHealth, digital health, precision mental health, idiographic analysis, person-specific modeling, p-technique, longitudinal study, precision interventions, smartphones, idiosyncrasy, psychological well-being, canonical correlation analysis, United States

## Abstract

**Background:**

Contrary to popular concerns about the harmful effects of media use on mental health, research on this relationship is ambiguous, stalling advances in theory, interventions, and policy. Scientific explorations of the relationship between media and mental health have mostly been found null or have small associations, with the results often blamed on the use of cross-sectional study designs or imprecise measures of media use and mental health.

**Objective:**

This exploratory empirical demonstration aims to answer whether mental health effects are associated with media use experiences by (1) redirecting research investments to granular and intensive longitudinal recordings of digital experiences to build models of media use and mental health for single individuals over the course of 1 year, (2) using new metrics of fragmented media use to propose explanations of mental health effects that will advance person-specific theorizing in media psychology, and (3) identifying combinations of media behaviors and mental health symptoms that may be more useful for studying media effects than single measures of dosage and affect or assessments of clinical symptoms related to specific disorders.

**Methods:**

The activity on individuals’ smartphone screens was recorded every 5 seconds when devices were in use over 1 year, resulting in a dataset of 6,744,013 screenshots and 123 fortnightly surveys from 5 adult participants. Each participant contributed between 0.8 and 2.7 million screens. Six media use metrics were derived from smartphone metadata. Fortnightly surveys captured symptoms of depression, attention-deficit/hyperactivity disorder, state anxiety, and positive affect. Idiographic filter models (p-technique canonical correlation analyses) were applied to explore person-specific associations.

**Results:**

Canonical correlations revealed substantial person-specific associations between media use and mental health, ranging from *r*=0.82 (*P*=.008) to *r*=0.92 (*P*=.03). The specific combinations of media use metrics and mental health dimensions were different for each person, reflecting significant individual variability. For instance, the media use canonical variate for 1 participant was characterized by higher loadings for app-switching, which, in combination with other behaviors, correlated strongly with a mental health variate emphasizing anxiety symptoms. For another, prolonged screen time, alongside other media use behaviors, contributed to a mental health variate weighted more heavily toward depression symptoms. These within-person correlations are among the strongest reported in this literature.

**Conclusions:**

Results suggest that the relationships between media use and mental health are highly individualized, with implications for the development of personalized models and precision smartphone-informed interventions in mental health. We discuss how our approach can be extended generally, while still emphasizing the importance of idiographic approaches. This study highlights the potential for granular, longitudinal data to reveal person-specific patterns that can inform theory development, personalized screening, diagnosis, and interventions in mental health.

## Introduction

Over the last decade, researchers have conducted extensive studies involving hundreds of thousands of participants to understand the relationship between media use and mental health. Despite significant investments, findings remain ambiguous, often reporting null or small associations [1-5]. This lack of clarity undermines progress in theory development, public policy, and clinical interventions. Researchers have proposed solutions, including granular tracking of media use [6-8], longitudinal study designs [9,10], the inclusion of at-risk populations [11-13], and person-specific analytics to account for individual differences [14,15].

A central challenge in this research is the problem of idiosyncrasy—the substantial variability in how individuals use media and how it affects their mental health [16,17]. In psychology, the opportunity to reclaim idiosyncratic variance has been defined as one of identifying idiographic filters for nomothetic interests [18]. The core argument is that the conventional practice of averaging out idiosyncrasy is neither theoretically satisfactory nor practically useful. We explore if and how an idiographic filter approach might enhance this research about media use and mental health. While researchers often seek universal rules to inform public policies or clinical interventions, such generalizations obscure the reality that individuals’ media experiences are inherently personal and shaped by unique combinations of content and context. Aggregate analyses, which average out these differences, are insufficient and often misleading. Similarly, mental health symptom clusters frequently cut across traditional diagnostic boundaries, further emphasizing the need for individualized approaches. Addressing this challenge requires an idiographic perspective, which preserves individual variability and analyzes person-specific patterns rather than treating them as noise. By adopting an idiographic approach, research can uncover meaningful insights into the unique configurations of media use and mental health for each person.

To address this challenge, we used p-technique canonical correlation analysis (pCCA) [19], a method designed to analyze within-person relationships over time. This approach creates idiographic filters [20] that highlight how core constructs, such as media use and mental health metrics, manifest differently for each participant. Unlike traditional methods that assume consistency across individuals, pCCA uncovers the specific patterns and dynamics unique to each person based on the common set of variables. For example, media use heavily characterized by frequent app-switching may correlate with symptoms of hyperactivity for 1 individual, while prolonged social screen time may have significant weight in describing the changes in symptoms of depression for another. By prioritizing individual-level insights, this method provides a pathway to more personalized interventions (Multimedia Appendix 1 [2-4,7,15-19,21-50]).

This study uses granular longitudinal data collected through the Human Screenome Project [21,51], which recorded smartphone activity every 5 seconds for 1 year, resulting in over 6.7 million screenshots and 123 surveys across 5 participants. Mental health metrics, including symptoms of depression [52], attention-deficit/hyperactivity disorder (ADHD) [22], state anxiety [53], and positive affect were assessed fortnightly. Using pCCA, we explored person-specific associations between media use and mental health to address the following questions.

- Can granular longitudinal recordings reveal associations between media use and mental health that differ from the null or small effects reported in aggregated studies?
- Can new metrics, such as fragmentation of media use, explain person-specific mental health effects and advance media psychology theory?
- Can combinations of mental health symptoms provide better insights into media effects than single measures of dosage or individual clinical symptoms?

By focusing on individual experiences and variability over time, this study offers a novel approach to understanding the relationship between media use and mental health, emphasizing the potential of person-specific data to inform theory development and precision interventions.

## Methods

### Procedure and Participants

This study was conducted in the United States, leveraging a longitudinal design to assess smartphone use and its relationships with mental health. Data were collected via a custom smartphone app and biweekly participant surveys, enabling the integration of behavioral data with mental health measures. Participants were recruited via the Qualtrics research panel as part of a larger research project on assessing digital experiences with smartphone data [51]. Eligibility criteria included residing in the United States, being the sole user of an Android phone, and providing informed consent and Health Insurance Portability and Accountability Act (HIPAA) authorization.

Participants installed a custom study app on their smartphones, enabling an unobtrusive collection of smartphone screens. The app required no active user management and operated transparently, with a status bar icon indicating its activity. Screenshots, along with operating system metadata like phone battery state and foreground app were captured and recorded automatically every 5 seconds when the screen was active.

Our study selected a focused sample that satisfied the minimum requirements for robust within-person analysis and allowed for a demonstration of our methodology, prioritizing long time series from a small number of participants over larger aggregate datasets so that we might uncover detailed person-specific insights and enable future scalability through computational methods [7,21,23]. We conducted a detailed analysis of 5 participants, randomly chosen from a group of 36 individuals that met several eligibility requirements. First, they had contributed at least 800,000 screenshots, reflecting an average of at least 3 hours of daily smartphone use over the year. This measure was chosen to capture use data that is close to the national average [54-56]. The goal was to ensure that participants had sufficient smartphone use to demonstrate relationships with mental health. Second, the selected participants had completed at least 20 biweekly surveys, creating a sufficient longitudinal record for each participant that exceeded the number of variables in the pCCA [57]. Finally, the selected participants had reported at least 1 biweekly period where their mental health symptoms reached clinically significant thresholds [22,52,58,59].

### Ethical Considerations

This study adhered to national regulations and institutional policies, including the Declaration of Helsinki (2013), and was approved by Stanford University’s institutional review board under protocol (#56430). All procedures were reviewed and classified as minimal risk.

Participants provided electronic informed consent and HIPAA authorization before enrollment. The consent process included detailed information about the study’s goals, procedures, and privacy protections, and participants confirmed their consent using an e-signature interface. To ensure privacy and confidentiality, all data were encrypted during transfer and securely stored on Stanford’s high-risk personal identifiable information and protected health information–compliant servers. Screenshots and metadata were deidentified prior to analysis, with access to raw screenshots restricted to a small subset of researchers under strict institutional protocols. Only aggregated, deidentified data were analyzed and reported to prevent the identification of individual participants. Participants were compensated US $15 for each fortnightly survey and data submission, with potential earnings of up to US $390 for completing the 1-year study.

Data were collected unobtrusively using a custom Screenomics app that captured screenshots every 5 seconds when the device was active. The app included an icon on the status bar to ensure transparency. Although nearly all screenshots were processed through automated methods, a small subset could be viewed by researchers in specific analyses. To mitigate risks, research staff were trained as mandated reporters under Stanford University and California guidelines. Any concerning content signaling potential legal, medical, or social risks was escalated to the Principal Investigators for appropriate action. Protocols were in place to refer participants to professionals or services as needed, ensuring their privacy while addressing potential risks to their well-being.

No images or supplementary materials in this manuscript include identifiable participant information. All visual data were anonymized to ensure privacy, and no consent was required for image inclusion as all images were deidentified.

All research staff completed mandatory HIPAA and human subjects training. Data security measures included strong passwords, restricted database access, and frequent reminders of privacy policies. These procedures ensured the highest level of data integrity and participant confidentiality.

### Measures

#### Mental Health Measures

Participants completed fortnightly surveys over a year to assess symptoms of depression, and ADHD, in addition to state anxiety and positive affect. This extended time frame allowed the study of fluctuations across weeks or months [24-29].

Depression symptoms were measured using the 10-item Center for Epidemiologic Studies Depression scale [52]. Participants rated the frequency of symptoms (eg, “I felt I could not shake off the blues”) on a 4-point Likert scale. Scores range from 0 to 60, with scores above 16 indicating clinical risk [59]. These scores were used to calculate the number of fortnight participants who were at clinical risk for depression (Table 1). The CES-D scale is in the public domain.

State anxiety was assessed with the 20-item State Anxiety subscale of the State-Trait Anxiety Inventory [60]. Items (eg, “I am tense; I am worried”) were rated on a 4-point Likert scale. Scores range from 20 to 80, with scores over 40 indicating moderate to high anxiety [58]. This threshold was used to calculate fortnights at clinical risk (Table 1). A license was obtained from Mind Garden, Inc.

Positive affect was measured using a single item: “Looking back over the last two weeks, I felt happy,” rated on a 100-point slider scale from “strongly disagree” to “strongly agree.”

Hyperactivity and inattention symptoms were measured fortnightly using the 18-item Adult ADHD Self-Report Scale [22,61]. Participants rated hyperactivity and inattention on a 5-point Likert scale. Subscale scores range from 0 to 36. A score of 9 or more on the first 6 items (4 inattention and 2 hyperactivity) indicates clinical risk for adult ADHD [62]. These thresholds were used to calculate risk fortnights (Table 1). A license was obtained from NYU TOV Licensing, New York University.

**Table 1:** Participant data and demographics^a^.

	Participant A (n=26)	Participant B (n=25)	Participant C (n=20)	Participant D (n=25)	Participant E (n=27)
Total screens	824,111	1,191,958	897,514	1,106,164	2,724,266
Age (year)	62	22	37	20	24
Sex	Female	Female	Female	Female	Female
Race	White	White	Multiracial Hispanic	White	Black
Region	Midwest	Midwest	Northeast	Pacific	Northeast
Residence type	Rural	Suburban	Suburban	Suburban	Urban
Highest educational level	2-year college	High school or GED^b^	Some college	Some college	Some College
Marital status	Divorced or separated	Single or never married	Married	Single or never married	Married
Income level (US $)	15,000-24,999	14,999 or less	25,000-29,999	75,000-99,999	14,999 or less

^a^The table presents the demographic characteristics of participants in the study, as collected through an initial onboarding survey. The survey was administered before the commencement of the study to gather pertinent information regarding participant backgrounds. “n” is the count of fortnightly surveys completed by each participant throughout the study year, while “Total screens” is the cumulative number of screenshots recorded from each participant’s smartphone during the study year.

^b^GED: General Educational Development.

#### Media Use Metrics

Six metrics for smartphone media use were derived from the screenshots and operating system metadata recorded continuously at 5-second intervals over the study year. Metadata enabled us to map app names and categories using Google Play Market classifications [63,64]. All metrics were averaged daily within biweekly periods, defined as the 14 days preceding each survey, except session duration calculated as the average screen-on interval. Missing data were addressed under the “missing at random” assumption.

Screen time was measured as the daily average number of screenshots captured during screen-on periods. The data collection app captured screenshots every 5 seconds when the screen was on. For each fortnight, the total screenshots were divided by the number of active data collection days.

Social screen time was measured as the daily average of screenshots recorded while using social apps. Social apps were those classified as “social” by Google Play Market categorization [63,64]. Social apps included common social media apps (eg, TikTok and Instagram), certain messaging apps (eg, Telegram and Text Free), and social network or dating apps (eg, Tagged and Grindr). Similar to the screen time measure, we calculated this metric by dividing the total number of social app screenshots by the number of active data collection days.

The number of sessions of smartphone use indicated average daily sessions, defined as intervals between screen on or off events. For each fortnight, we calculated the daily average number of sessions by dividing the total number of sessions by the number of days the participant actively used their phone. A higher number of sessions (eg, ten 30-second sessions) relative to the duration of each session (eg, one 5-minute session) indicates a more fragmented digital experience.

Session duration was calculated as the time interval between the initiation of a session and its termination. For each fortnight, we summed the duration of all sessions that occurred within that period and then computed the mean. This provided an average session duration metric for the specified time frame.

The number of unique apps was calculated as the daily average of distinct apps used, calculated by dividing the count of unique apps within a period by the number of active days.

The number of app switches indicated the daily average number of times individuals switched between apps and was calculated as a measure of fragmentation. For each biweekly period, we counted how many times individuals moved from app to app (ie, from Facebook to Zoom=1 switch) and divided that number by the number of active smartphone data collection days. Please see Multimedia Appendix 1 [2-4,7,15-19,21-50] for more discussion on theories and measures examining media use and mental health over time and the origins of our measures.

### Data Analysis

Canonical correlation analysis (CCA) is a classical technique used to obtain the maximal correlation between objects that are represented by 2 sets of variables [65]. Adopting the Nesselroade et al idiographic filter approach [18], we use pCCA to model each individual’s multivariate repeated measures data. The observed correlation between an individual’s media use and mental health is not static; rather, it depends on the chosen coordinate system, that is, how observed variables are projected onto latent constructs of media use and mental health. Unlike traditional factorial analyses that assume a uniform variable projection across individuals, the idiographic filter approach allows person-specific rotations of the coordinate system.

pCCA does this by locating the specific rotation where the linear correlation between the latent media use and mental health canonical variates is mutually maximized. The size and significance of the maximum correlation (ρ) are used to answer our first research question: Does the analysis of single individuals offer different conclusions than the small or null effects reported in the across-individual analyses dominating the literature? The configurations of the basis vectors *x* and *y* are interpreted to address our second and third questions: Can we identify combinations of media use metrics and mental health metrics that might advance theories about media effects?

Given *A_x_* (27 occasions × 6 media use variables) and *A_y_* (27 occasions × 5 mental health variables) for an individual, the data are projected onto canonical variates, *z_x_ = A_x_x* and *z_y_ = A_y_y*, the correlation of which is given by the following equation.



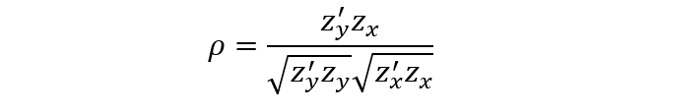



where *x* and *y* are chosen to maximize ρ, achieved via singular value decomposition [19,66]. We use permutation tests to ensure observed correlations are not simply artifacts of random chance. Specifically, for each participant, we perform 1000 permutations by randomly shuffling the values within the columns of *A_x_* or *A_y_*, disrupting any temporal ordering between media use and mental health. This process generates a null distribution for ρ, representing the range of chance correlations expected in the absence of a systematic relationship. Comparing the observed ρ to this null distribution provides an exact *P* value based on the empirical data, thereby avoiding reliance on theoretical distributions that could inflate *P* values. Histograms of these permutations are presented in Multimedia Appendix 2. All pCCA and permutation test analyses were done using the *cca* package [67] in R (R Foundation for Statistical Computing).

## Results

The following results include an analysis of a full year of media use and mental health data (6,744,013 screenshots and 123 fortnightly surveys) provided by 5 individuals. The background of each participant and descriptive statistics about media use and mental health are in Tables 1-3.

**Table 2:** Descriptive statistics for participants' mental health measures throughout 1 year^a^.

Participants	Depression symptoms (0-60)			State anxiety (20-80)				ADHD^b^ symptoms (0-72)							Positive affect (0-100), mean (SD)
	Mean (SD)		Clinical risk periods, n	Mean (SD)	Clinical risk periods, n			Inattention (0-36), mean (SD)		Hyperactivity (0-36), mean (SD)		Clinical risk periods, n			
A	9.23 (5.31)	1		44.23 (4.43)		22	1.88 (3.24)		2.04 (3.32)		0		53.85 (17.54)		
B	24.80 (5.07)	24		51.84 (10.15)		21	12.72 (1.99)		8.72 (1.79)		13		62.12 (19.81)		
C	12.40 (4.77)	4		45.05 (14.25)		13	10.10 (2.25)		5.70 (0.80)		3		65.35 (5.43)		
D	12.80 (6.10)	7		45.84 (8.76)		17	5.36 (2.27)		2.76 (1.39)		0		57.00 (15.21)		
E	14.78 (7.06)	4		55.00 (8.98)		27	4.52 (4.37)		4.34 (4.36)		0		16.85 (27.98)		

^a^The table shows the means and SD for each mental health measure, as well as the clinical risk associated with symptomatology exceeding certain thresholds. Clinical risk periods are calculated as the number of fortnightly surveys in which participants’ responses exceeded the clinical threshold for each measure.

^b^ADHD: attention-deficit/hyperactivity disorder.

**Table 3:** Descriptive statistics for participants' media use measures throughout 1 year.

Participant	Screen time (hours per day), mean (SD)	Social screen time (hours per day), mean (SD)	Number of sessions (per fortnight), mean (SD)	Session duration (minutes per day), mean (SD)	Number of unique apps (per day), mean (SD)	Number of app switches (per day), mean (SD)
A	2.99 (0.51)	0.41 (0.17)	57.84 (8.12)	3.10 (0.49)	4.08 (0.39)	210.27 (32.79)
B	4.15 (1.10)	0.37 (0.12)	32.76 (3.18)	7.59 (1.86)	3.64 (0.37	206.87 (18.96)
C	4.18 (0.95)	0.15 (0.23)	57.81 (8.89)	4.33 (0.85)	7.29 (2.50)	324.88 (52.73)
D	4.18 (1.05)	0 (0)	90.90 (25.28)	2.87 (0.89)	4.54 (0.61)	273.22 (54.38)
E	9.55 (2.03)	0 (0)	9.45 (2.10)	62.62 (14.75)	1.50 (0.59)	39.51 (14.48)

The main objective of this study was to assess whether analysis of media use metrics derived from super-intensive observational data passively collected from the smartphones individuals use in their daily lives could unearth relationships between media use and mental health within individuals that have previously been difficult to observe using analyses aggregated across individuals. Related to that objective, we first report the within-person associations between the media use and mental health latent variables that were obtained separately for each participant using pCCA.

**Figure 1:**
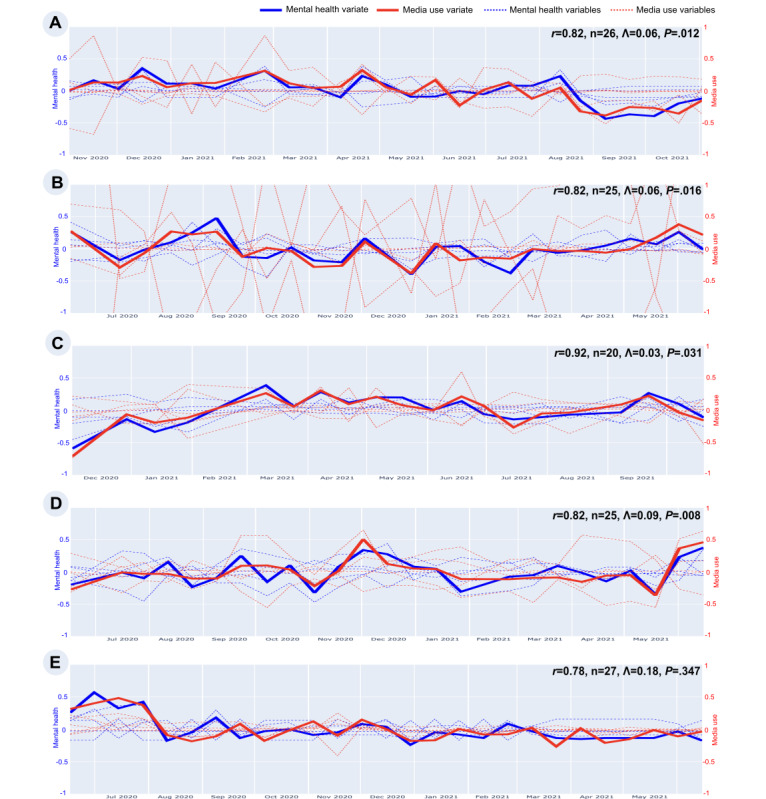
Canonical variate plot for each participant (A-E). This figure presents the results of pCCA, highlighting within-person associations between media use and mental health metrics for five female Android phone users in the United States. The data were collected longitudinally as part of the Human Screenome Project, spanning 1 year (from July 2020 to October 2021). For each participant, the figure shows the temporal alignment of canonical media use variate (red solid line) and mental health variate (blue solid line), derived from granular measures recorded fortnightly. Individual metrics of media use (dotted red lines, eg, app-switching frequency, social screen time) and mental health (dotted blue lines, eg, depression, state anxiety) are also plotted in the background for comparison. Canonical correlations (r) for each participant range from 0.78 to 0.92, indicating strong covariation. Wilks statistic (Λ), *P* values, and sample size (n) are displayed for statistical significance. Permutation tests were conducted to validate correlations by generating null distributions based on shuffled data. Participant A shows a strong positive association between mental health and media use, with both variates generally moving together over time. Participant B exhibits a similar pattern, though with more pronounced fluctuations. Participant C has the highest correlation, indicating tightly coupled trends in media use and mental health across the year. Participant D also shows a strong association, though with some deviations at certain time points. In contrast, Participant E presents a weaker and statistically non-significant correlation, suggesting that media use and mental health follow more independent patterns in this case. These findings illustrate the unique within-person dynamics of media use and mental health over time, emphasizing the importance of person-specific models in understanding this relationship.

The main results of this study are reported in Figure 1, which shows correlations and plots of the fortnightly media use and mental health variables across 1 year for each participant. The observed p-technique canonical correlations are large, ranging from 0.78 to 0.92. These values provide a compelling answer to our primary research question: for 4 of the 5 participants in this study, fluctuations in canonical media use variate were significantly and tightly coupled with fluctuations in a canonical mental health variate, more so than for any previous associations observed in this literature [3,5].

The composition of media use and mental health variates was unique for each participant, reflecting person-specific relationships and highlighting the heterogeneity in how media use impacts mental health. While individual metrics (eg, app-switching, screen time) varied considerably over time, the canonical variates showed consistent and strong covariation for four of the 5 participants. These findings underscore the use of pCCA in identifying tightly coupled, within-person associations that are otherwise obscured in aggregated analyses, paving the way for more individualized theoretical insights and interventions.

The figures below highlight the unique person-specific relationships between media use metrics and mental health measures. These findings demonstrate the variability in how media use correlates with mental health for each participant, emphasizing the value of individualized approaches.

**Figure 2:**
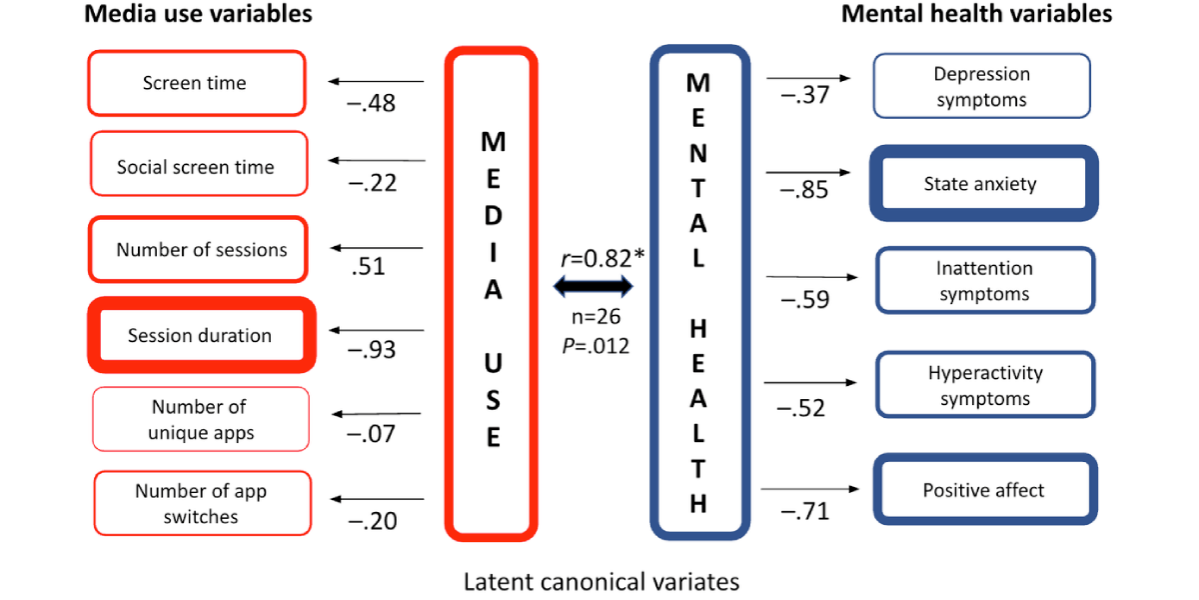
Canonical correlation analysis of participant A’s media use and mental health. This figure illustrates the results of pCCA for participant A, based on 1 year of longitudinal data. Media use (left column) includes 6 variables (eg, session duration, number of sessions, and screen time), while mental health (right column) includes 5 variables (eg, state anxiety, depression symptoms, and positive affect). Arrows from the canonical variates (center) to observed variables indicate the direction (positive or negative) and strength of associations, with values closer to ±1.0 indicating stronger relationships. The canonical correlation (*r*=0.82; *P*=.01) highlights a significant within-person relationship, showing that quick-checking behaviors (eg, shorter session duration and more frequent sessions) were associated with reduced anxiety and ADHD symptoms but also diminished positive affect.

For participant A (Figure 2), the media use behavior most related to greater mental health symptoms over time was primarily shorter sessions (–0.93), and secondarily a higher number of sessions (0.51) and shorter screen time (–0.48). This suggests that the fluctuations in media use most related to mental health for this person are quick-checking behaviors on the smartphone. In complement, the configuration of mental health symptoms and positive affect that was most related to fluctuations in media use was characterized by lower state anxiety (–0.85) and lower levels of ADHD symptoms for both inattention (–0.59) and hyperactivity (–0.52), and lower positive affect (–0.71). Generally, for participant A, quick checking on the smartphone was associated with lower positive affect, and lower state anxiety and ADHD symptoms.

**Figure 3:**
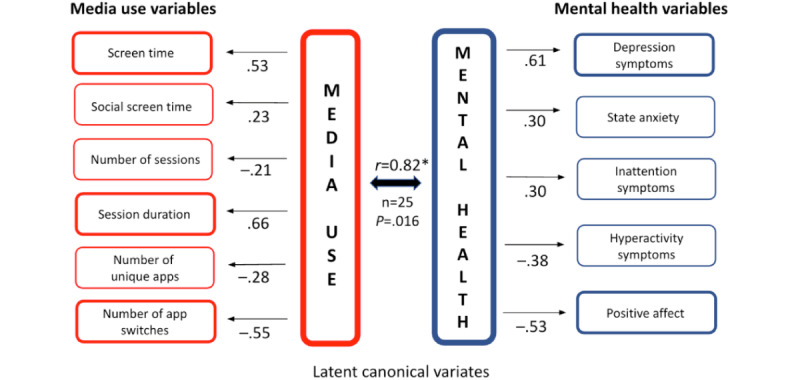
Canonical correlation analysis of participant B’s media use and mental health. This figure illustrates the results of pCCA for participant B, based on 1 year of longitudinal data. Media use (left column) includes 6 variables (eg, session duration, screen time, and app-switching intensity), while mental health (right column) includes 5 variables (eg, depression symptoms, state anxiety, and positive affect). Arrows from the canonical variates (center) to observed variables indicate the direction (positive or negative) and strength of associations, with values closer to ±1.0 indicating stronger relationships. The canonical correlation (*r*=0.82; *P*=.02) highlights a significant within-person relationship, showing that focused smartphone use—characterized by longer session duration, higher screen time, and minimal app-switching—was associated with increased depression symptoms and reduced positive affect.

Figure 3 shows that for participant B, the media use behaviors most related to greater mental health symptoms over time were characterized by longer sessions (0.66) and secondarily by more screen time (0.53) and little app switching (–0.55). We characterize this combination as focused smartphone use. The mental health symptoms most related to media use were characterized by depression symptoms (0.61) and lower positive affect (–0.53). It is also notable that this participant’s mental health changes, especially true for depression symptoms, had a stronger association with media use changes than for state anxiety. The media use canonical variate for participant B was driven by fluctuations in total screen time rather than social app use, specifically.

**Figure 4:**
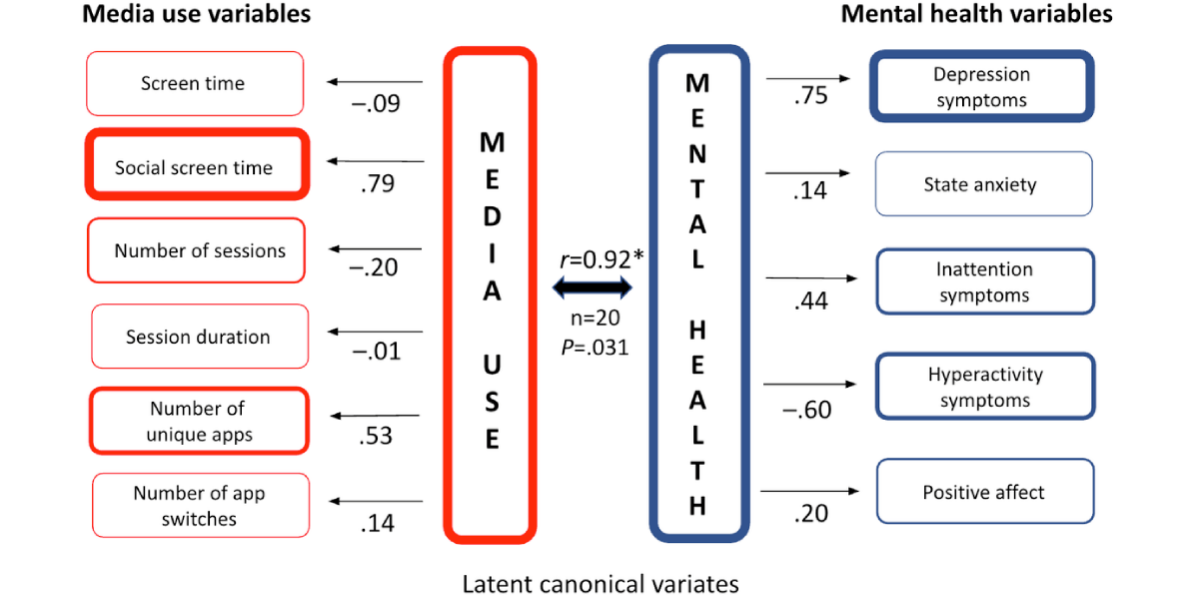
Canonical correlation analysis of participant C’s media use and mental health. This figure illustrates the results of pCCA for participant C, based on 1 year of longitudinal data. Media use (left column) includes 6 variables (eg, social screen time, number of unique apps, session duration), while mental health (right column) includes 5 variables (eg, depression symptoms, hyperactivity symptoms, and positive affect). Arrows from the canonical variates (center) to the observed variables indicate the direction (positive or negative) and strength of associations, with values closer to ±1.0 reflecting stronger relationships. The canonical correlation (*r*=0.92; *P*=.03) underscores a significant within-person relationship, with social screen time (0.79) emerging as the strongest media use factor, closely tied to increased depressive symptoms (0.75) and reduced hyperactivity symptoms (–0.60).

For participant C (Figure 4), the media use canonical variate shows the strongest loadings for the number of social screen time (distinct from total screen time) at 0.79. Secondarily, there is a high loading for the use of unique apps (0.53). The mental health canonical variate shows a strong loading associated with depression symptoms (0.75). Additionally, there is a noteworthy negative loading in relation to hyperactivity symptoms (–0.60). The much larger loading for social screen time in comparison to total screen time shows the value of separating these uses when examining relationships with mental health. This distinction is often part of discussions about media effects (ie, more worry about intensive social app use and depression than about the effects of all media use). As for many media effects, however, the value of the distinction is person-specific.

**Figure 5:**
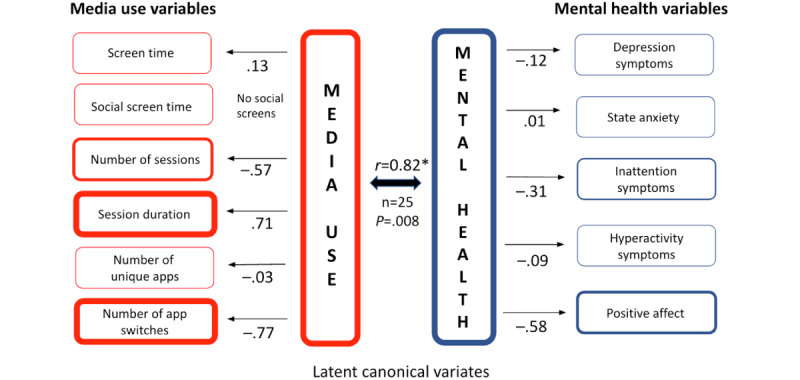
Canonical correlation analysis of participant D’s media use and mental health. This figure presents the results of pCCA for participant D, based on longitudinal data collected over 1 year. Media use (left column) includes 6 variables (eg, session duration, number of sessions, and app switching), while mental health (right column) includes 5 variables (eg, positive affect, state anxiety, and ADHD symptoms). The arrows between latent canonical variates (center) and observed variables represent the direction (positive or negative) and strength of associations, with values closer to ±1.0 indicating stronger relationships. The canonical correlation (*r*=0.82; *P*=.008) underscores a significant within-person association, highlighting that longer session durations and reduced app-switching behaviors were most associated with lower positive affect for participant D.

Participant D (Figure 5) did not use any social apps during the entire year (ie, there are no screens from any of the hundreds of social apps in the Google Play store). Their media use canonical variate is characterized by fewer app switches (–0.77), extended session durations (0.71), and a reduced number of sessions overall (–0.57). This pattern indicates longer, focused interactions. The mental health canonical variate was characterized primarily by reduced positive affect (–0.58), with no large loadings associated with any of the mental health symptoms. For participant D, longer and more focused smartphone sessions had a more singular definition (diminished positive affect). However, the number and duration of the sessions were still highly correlated with smartphone use features (*r*=0.82; *P*=.008).

**Figure 6:**
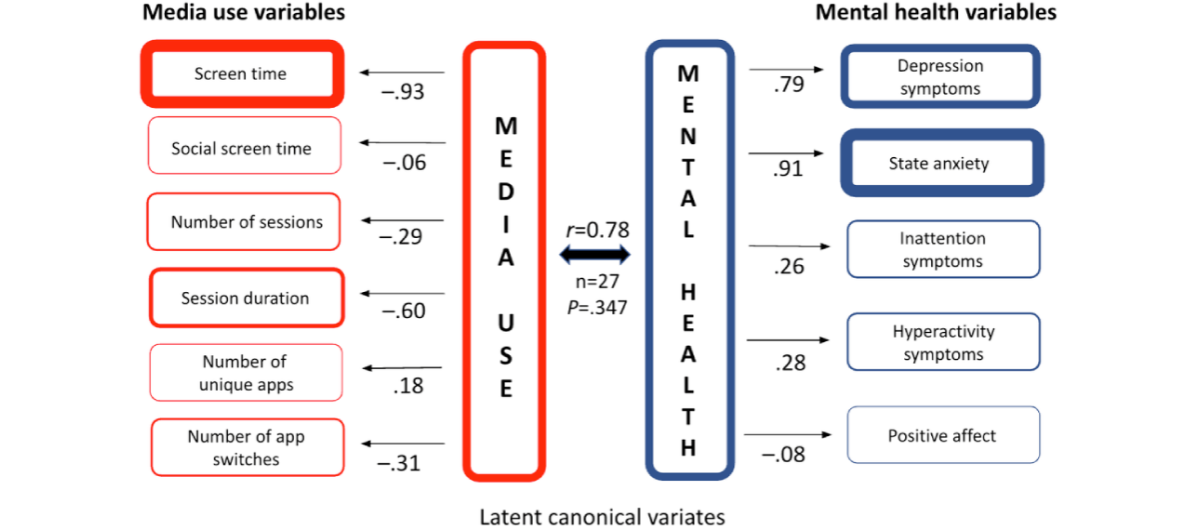
Canonical correlation analysis of participant E’s media use and mental health. This figure illustrates the results of pCCA for participant E, based on 1 year of longitudinal data. Media use (left column) is represented by 6 variables, such as screen time, session duration, and number of app switches, while mental health (right column) is represented by 5 variables, including depression symptoms, state anxiety, and positive affect. Arrows connecting latent canonical variates (center) to observed variables indicate the direction (positive or negative) and strength of associations, with values closer to ±1.0 reflecting stronger associations. The canonical correlation (*r*=0.78; *P*=.35) indicates a nonsignificant association.

There was no statistically significant association between the media and mental health canonical variates for Participant E (Figure 6).

## Discussion

### Principal Findings

The results highlight the person-specific associations between media use and mental health. Canonical correlations revealed substantial covariation (0.82 to 0.92) over time for 4 out of 5 individuals. Simultaneously, as hypothesized, the specific combinations of media dosage metrics and dimensions of mental health that defined this covariation were different for each person (Multimedia Appendix 1 [2-4,7,15-19,21-50]. The approach supports the idea of a common association between media use and mental health while accommodating idiosyncrasy in how that association manifests for individuals. The couplings between media use and mental health for each person offer insights into the complexity of this relationship, directly addressing the research questions posed in the introduction.

In summary, our conclusions are the following.

- As research investments shift toward intensive, granular longitudinal data about individuals’ digital experiences, this study demonstrates the potential to construct distinct, person-specific models of media use and mental health, revealing insights that transcend traditional aggregated analyses.
- The study’s innovative approach, combining various dosage metrics of fragmented media use, advances person-specific theorizing in media psychology, offering potential explanations for the impact on mental health and emphasizing the significance of tailored approaches.
- By identifying individualized combinations of mental health symptoms, the study underscores the necessity of considering nuanced factors beyond single measures of dosage and affect or clinical symptoms associated with specific disorders.

### Causality and Theory

The substantial p-technique canonical correlations observed in this study reflect within-person associations between media use and mental health but do not establish causality. Media use metrics may act as markers responding to mental health changes, antecedents influencing mental health, or reflections of confounding variables. A causal explanation likely involves complex mechanisms interacting over different time domains and individual histories, which this analysis does not aim to disentangle.

Past research has explored single mechanisms over varying time frames, such as the introduction of Facebook on campuses (months/years) [68], ecological momentary assessment studies linking self-reported media use with mental health (days/weeks) [69], and cohort analyses over decades [70]. Additionally, momentary exposures, like a single unflattering social media post, might amplify depression symptoms, particularly during periods of heightened vulnerability. Interactions between short- and long-term domains—for example, a negative social media post during an extended depressive episode—could further complicate causal pathways.

Combining mechanisms into a single causal conclusion for media use and mental health is beyond the scope of this project and likely any single study, given the heterogeneity observed across individuals. The participants in this study suggest that no universal explanation applies across diverse media behaviors, content, and time domains. Instead, multiple mechanisms, each tailored to specific contexts, are likely needed. Controlled experiments targeting distinct media experiences [71,72] and idiographic approaches like ours can help identify mechanisms for further exploration.

The within-person associations in this project emphasize the need for new theories addressing the accumulation of similar media experiences over time, regardless of specific content. Such mechanisms echo the concept of resonance in social reality theory [73,74], where outcomes arise from the alignment of digital and real-life environments. For instance, increased screen use during a depressive episode may reinforce social isolation, creating a feedback loop that intensifies mental health symptoms [75]. These findings highlight the importance of reexamining how repetitive media experiences influence mental health over extended periods.

### Interventions

The p-technique canonical correlations observed in this project (Figure 1) suggest that results obtained for a participant over 1 year may generalize to future months. Acute changes in media use could serve as markers for triggering mental health interventions without requiring frequent or intrusive direct assessments. Interventions could integrate patient and clinician access to data through dashboards alerting users to mental health risks and offering tailored recommendations. For instance, systems might limit social app usage for some individuals or promote it for others based on identified risks. Person-specific thresholds for intervention provide a more accurate approach than generalized thresholds derived from population averages [76].

The key to these interventions is precision timing and targeted actions that connect continuous monitoring of screen activity with information designed to improve mental health. Screen-based systems might rival the diagnostic accuracy of traditional methods for some disorders. Studies estimate, for example, that up to 60% of people with depression are never diagnosed [77,78], and even those connected to care often face delays in appointments, diagnosis, and treatment initiation [79]. Screen summaries might accelerate care by offering behavioral insights prior to intake or as indicators of treatment effectiveness.

Determining the clinical use of idiographic tracking will require research to establish optimal baseline collection periods, which may vary by individual. While longer data collection can enhance accuracy, even shorter periods could yield valuable insights. For example, smartphone monitoring during the weeks between initial clinician contact and the first appointment, or in critical postdischarge periods, could inform preliminary evaluations and adaptive interventions. These monitoring systems could feed data into clinician dashboards and support the implementation of just-in-time adaptive interventions [80]. Importantly, such tools are intended to augment rather than replace direct patient-clinician interactions, which require robust support resources.

### Limitations of the Study

This longitudinal study, conducted with a small, nonclinical sample of female Android phone users, was not intended to provide population-level conclusions about the prevalence of media and mental health relationships nationally or within subgroups. Avoiding generalization at this stage helps circumvent the small sample fallacy [81]. Our intent was to first understand the unique media dosage and mental health indicators important for individuals without prematurely investing in a research strategy aimed at extensive between-subject variability that we believe has resulted in considerable ambiguity in this literature. This bottom-up inductive approach celebrates idiosyncrasy and prioritizes individual models as a first step toward building models that might apply to larger groups of people. We have successfully demonstrated that it is possible to find strong, individualized relationships with granular measures and longitudinal data.

Our analysis focused on a limited set of variables: 6 media use metrics and 5 mental health measures. Many additional factors could influence within-person associations between media use and mental health. Notably, data collection coincided with the height of the COVID-19 pandemic. Although none of the participants contracted the virus, the pandemic’s broader effects may have shaped their mental health and media use behaviors. Future analyses could examine how contextual factors, such as global or personal life events, moderate these relationships. For example, exploring interactions between media use, sleep patterns, and mental health outcomes [82,83], or identifying whether media use acts as a supportive resource [84,85] or an additional stressor [86,87] could inform context-aware models that better capture individual contingencies.

### Future Work and Conclusions

While new computational methods enable the collection and analysis of large datasets, they often focus on aggregated measures, obscuring person-specific longitudinal insights and yielding ambiguous population-level conclusions. Our method emphasizes the importance of a person-specific approach applicable to new datasets. A key recommendation for extending this investigation is to maintain this individual focus and compare people based on similarities between models derived for single individuals, rather than relying solely on priori groupings based on demographics or other psychosocial characteristics. When idiographic models are available for many individuals, researchers can use clustering, latent class, and mixture models to quantify and examine between-person similarities and differences [88-90]. One such method suggested by Ram et al [88] involves calculating Tucker congruence coefficients [91] that compare parameter estimates (eg, the canonical variates) for each pair of individuals in a larger sample. Importantly, this approach entails specifying and modeling each person’s data separately, as done in this study, before identifying shared patterns. Rather than assuming a priori commonalities (eg, based on sex or personality), this bottom-up paradigm uncovers the full range of person-specific solutions, which can then inform theory, policy, and interventions [18,88,92,93].

Future work will expand on the metrics presented here, integrating richer variables through our computational assay [20] and identifying screen content characteristics—such as words, objects, emotional arousal, and environmental features—using advanced artificial intelligence models. These granular data can help refine our understanding of how different media use patterns related to mental health. While traditional screen time measures, often derived from self-reports, have been criticized for their imprecision [94], this study suggests that accurate, granular recordings of screen behavior can reveal strong associations for some individuals. Similar increments in the precision of assessing media use have paid off in the study of other media effects; for example, exposure to political information [95], advertising [94], and food content in digital media [30]. Our ongoing work also includes open-sourcing the most up-to-date Screenomics app and toolset [96].

The idiographic filter approach, producing individual models 1 person at a time, may initially seem to risk overfocusing on small samples. However, there may not be a shorter route to useful answers about media use and mental health. This is especially true if we want to retain media use and mental health as important and psychologically complex categories of digital and non-digital life. The present results suggest that to advance research addressing media use and mental health problems generally, we may need to seek answers applicable to one person at a time; that is, we must embrace idiosyncrasy, not ignore it.

## Appendix

Multimedia Appendix 1 Supplementary Information.

Multimedia Appendix 2 Permutation distribution histograms. This figure displays histograms of the permutation distributions of 1,000 permuted canonical correlations for each participant (A to E). To assess the statistical significance of the observed canonical correlations, we conducted permutation tests using Wilks' lambda by randomly permuting each individual’s data, creating a null distribution. This null distribution represents correlations expected by chance—meaning the values that would arise if there were no systematic relationship between media use and mental health variables and any observed associations were purely random. The histograms display these chance-based distributions under the null hypothesis, with observed canonical correlations indicated by red dashed lines to evaluate their statistical significance. The test statistic shown in each histogram is Wilks' lambda. For Participant E, the result is nonsignificant (*P*> 0.05), suggesting that the observed correlation cannot be confirmed as statistically significant. For the other participants, the observed correlations are confirmed by the permutation test.

## Abbreviations

ADHD: attention-deficit/hyperactivity disorder
CCA: canonical correlation analysis
HIPAA: Health Insurance Portability and Accountability Act
pCCA: p-technique canonical correlation analysis

## Data Availability

All deidentified data used and reported in this study, along with the code for analysis and visualizations, are available from the corresponding author upon reasonable request.
