# Supplementary material for: Person-Specific Analyses of Smartphone Use and Mental Health: Intensive Longitudinal Study
Source: JMIR Form Res. 2025 Feb 26;9:e59875. doi: 10.2196/59875 (PMC11904378; doi:10.2196/59875)
Supplement: Multimedia Appendix 1 [file formative_v9i1e59875_app1.docx]

## The Problem of Idiosyncrasy

The problem of idiosyncrasy may be one of the thorniest challenges to research about media use and mental health; a problem voiced over the decades about media effects research generally [16,17]. Addressing this problem head-on, we adopted an *idiographic approach* that embraces and preserves individual variation. In psychology, the opportunity to reclaim idiosyncratic variance has been defined as one of identifying *idiographic filters* for nomothetic interests [18]. The core argument is that the conventional practice of averaging out idiosyncrasy is neither theoretically satisfactory nor practically useful. We explore if and how an idiographic filter approach might enhance current research about media use and mental health.

The idiosyncrasy problem is a tension between the desirability for general rules that apply to everyone and the reality that there are individual variations that manifest as exceptions to the general rules. We note three dimensions of this tension that are important for studying media use and mental health. First, we recognize that individual idiosyncrasy can be an anathema to societal-level conclusions (e.g., government laws and regulations, parent advisories, community medical or public health interventions, and policies) that can hopefully be addressed with universal rules. That search for universality, however, diverts attention from a critical reality of media effects – each person uses and responds to media differently. Second, idiosyncrasy exists in part because people weave fragments of media into wholes that are thoroughly personal, essentially creating a situation where the stimuli are unique for each user [7,75,77]. Because each individual’s media exposures are unique, any aggregations needed to discover universals are potentially meaningless in that they represent no one’s media. Third, there is increasing recognition of idiosyncrasy in mental health that has focused research on symptom clusters that cut across standard definitions of mental health disorders [78]. Examples of person-specific treatments and interventions include personalized models for the prediction of suicidal ideation [79,80], the treatment of panic disorders [81], the diagnosis of adult ADHD [24], and the heterogeneity of depression in adolescents [82].

## Idiographic Filters: A Person-Specific Analytic Strategy

There is a growing interest in analytics that can accommodate individual idiosyncrasies within a common set of concepts. In response, we extend traditional canonical correlation analysis (CCA) [19,83] into a p-technique canonical correlation analysis (pCCA) that allows us to identify how core constructs, ones that we believe are generally applicable *across* individuals, manifest for a given individual. Using pCCA, we analyze a common set of observed variables—specific aspects of media use and mental health—collected for each individual over one year. This analysis projects these observed variables onto new latent variables (canonical variates of media use and mental health), while optimizing the weights of the observed variables to construct canonical variates with the highest correlation (see Methods). This strategy acknowledges that classical factorial invariance cannot be assumed across participants. Instead, the approach allows us to discover the specific response patterns through which each individual’s unique configuration of media use and mental health manifest on their smartphone and in their daily life.

Our use of the idiographic filter approach (pCCA) naturally invokes a tradeoff between total number of participants studied and the number and range of observed time points for each participant. We purposively prioritize obtaining and analyzing long time series from a few individuals. Following examples set by genomics, personalized medicine, and *in situ* studies of digital behavior [7,21,26], the rationale being invoked is that studies should not be judged as useful or misleading because they make inferences about five rather than 5,000 people. Instead, they should be considered useful in relation to the detail they provide about *any* single person’s experiences. We similarly propose that intensive tracking of individuals might open possibilities for understanding aspects of media use as predictors, markers, and potential causes of mental health symptoms. Moreover, when this idiographic approach is demonstrably successful, today’s computational technologies provide possibility for it to be scaled relatively easily [7,21,26].

## Examining Media Use and Mental Health Over Time: Theories and Measures

The extended time period for this study is critical for studying the course of changes in both media use and symptoms of mental health. The year-long period represents a major shift from the typical investigation of cross-sectional between-person differences assessed at one moment in time to longitudinal serial assessments that detect within-person fluctuations. While not emphasized in the media psychology literature, there are known individual differences in media use over days, weeks, and seasons [4,33]. Similarly, symptoms of target mental health disorders are known to vary substantially within people over time. This is true, for example, for periods of mania [34,35], depression [36], and anxiety [37] where symptoms may build or resolve over several months, and also for changes in positive aspects of well-being that may vary considerably over similar months-long time periods [38]. Hence, through analysis of media use data obtained over a time period in which mental health symptoms change (i.e., at least one year), research can observe natural fluctuations that may be caused, accelerated, or slowed by media use.

### Media Use as Manifestations and Triggers of Mental Health Symptoms

The largest literature about media use and mental health concentrates on well-being [3,84]. Definitions of well-being are numerous but center around evaluations of happiness, sadness, acceptance by others, loneliness, worry, anger, and stress [85]. There is research, for example, that creates indices that mix well-being (e.g., happiness) with ill-being (e.g., depression and anxiety) [86]. Most summaries, however, separate well-being and health disorders, finding no relationship or small positive relationships for both, and the studies note considerable variability in reported associations [15,87,88].

Prior literature about media effects related to mental health symptoms is more sparse than literature about general well-being. Studies in these areas, including those mentioned in comprehensive and systematic reviews [2,3], have revealed mixed effects, and no clear consensus about the impact of media on mental health disorders. This ambiguity persists despite several theoretical proposals about media use as a sign or cause of mental health disorders.

In this study, mental health metrics were chosen to span a range of indicators [89], including symptoms of psychiatric disorders (i.e., depression and ADHD), state anxiety, and self-reported positive affect. There has not been a consensus in the literature about the most important dimensions of mental health to examine. Consequently, most reviews of media use and mental health cover studies that span several aspects of health and well-being [87,90,91]. Thus, we chose a range of measures to test how multiple elements of dosage might be uniquely related to multiple mental health symptoms for each participant.

### Screen Dosage and Theories About Media Effects

This section reviews the conceptual origins of our media use measures and anticipates how these measures might contribute to person-specific theoretical models that help explain how smartphones are related to mental health. Almost all theories about media influence make assumptions about how variability in media exposure —or dosage— affects individual thoughts and behaviors. The simplest and most common dosage concept, and the one that covers the vast majority of the media literature, is defined as the *quantity of time* that a person spends experiencing media; for example, hours per day using Instagram, minutes per day reading a newspaper, number of movies watched during a week. However, there are many other possible ways to capture the variability of dosage of media exposure, and each could be useful for advancing theory. Consequently, a theoretical goal of this project is to define multiple components of dosage, borrowing from similar conceptions in medicine [92] to develop measures that better inform theories about smartphones and mental health.

We have labeled four different characteristics of dosage: 1) total quantity, 2) frequency, 3) duration per use, and 4) intensity of activity during use. In our study, *total quantity* refers to the total amount of smartphone exposure over a given period of time. *Frequency* refers to how often smartphone sessions are initiated during that period. *Duration* per use refers to the length of time the smartphone is on during each session (e.g., 5 seconds vs. 5 minutes). *Intensity of activity during use* measures the level of involvement, defined by how much manipulation and app-switching occurs per unit of time, similar to prior concepts of interactivity [93]**.**

The vast majority of studies about media and mental health examine between-person differences in the *quantity* of time spent with media and mental health symptoms [3]**.** These other dosage elements (frequency, duration, and intensity) are less studied, in part because they have been more difficult to measure. With advances in data logging and computational methods, however, these components are more accessible. Our project uses six metrics of dosage linked to these three elements, each captured using the Screenomics framework that samples individual screens in addition to operating system metadata. For *quantity*, we measured the total number of screens viewed, the total number of social screens viewed, and the number of unique apps used in a given time period. *Frequency* was measured by the number of sessions recorded per day; *duration* by the average time of each session per day; and *intensity* by the number of app switches (i.e., interactivity) per day during a given time period.

Our theoretical goal was to examine how, for a given individual, fluctuations in the different aspects of dosage are related to fluctuations in mental health. Currently, theory in this area emphasizes the mechanisms of mental health changes, without corresponding detail about how different elements of smartphone use change over time. There are valuable new attempts to link mental health outcomes to specific affordances of smartphones, particularly social media [94]; however, specific links to different dosage elements of media use are still rare.

Our general hypothesis is that media use metrics will covary with mental health metrics, but the prioritization and even the direction of these relationships may be unique for any given individual. The four dosage elements could help elaborate on how various theories might be modified to apply to specific individuals. For example, consider theory about the influence of smartphones on self-concept [95]. Theories in this area suggest that beliefs and evaluations about ourselves are shaped by smartphone interactions, including the time spent on devices, the reception of feedback from others (especially negative evaluations), and the amount of social content consumed. However, while the quantity of time spent on smartphones or social media is often considered, the specific dosage elements of that time (such as frequency or intensity) remain underexplored.

These dosage elements can help clarify specific theoretical questions around self-concept and smartphone use. For example, separating total screen time from social screen time could determine whether self-concept changes are primarily tied, as most speculate, to social critiques aimed directly at the user, or alternatively, whether mental health outcomes are influenced by more generalized experiences, such as exposure to community or world events that are not personalized but conveyed through more general content. By assessing frequency—measured by daily smartphone sessions—alongside quantity, research could explore, for example, whether social media experiences require regular reinforcement throughout the day, or whether isolated but intensive single exposures have lasting effects. This consideration of whether smartphone influence has a “half-life” opens up new avenues for exploring whether the effects of social media diminish over time or persist even if not constantly reinforced.

Longitudinal analysis of frequency, duration, and intensity can also contribute to theorizing around habitual smartphone checking, which has been shown to heighten reward sensitivity over time [94] and lead to more fragmented smartphone use [96]. Short duration and high intensity, reflected in frequent app-switching and smartphone checking, may engage the brain’s reward system, reinforcing checking behavior. By examining how these elements of dosage affect reward-driven interactions, we can gain deeper insights into how habitual media use behaviors evolve and contribute to person-specific mental health symptoms.

The use of dosage elements to inform theory is exploratory. Assuming that our p-technique analysis can identify patterns for individuals, these dosage elements will be useful for building person-specific theories that are more closely aligned with media use on smartphones—many of which may not yet be represented in the current literature. In the person-specific models reported in the results, we show how the fluctuations over time in these different aspects of dosage are related to fluctuations in mental health, offering a novel approach to theorizing about the relationship between media use on smartphones and mental health.

## The Concept of Screen Dosage

We proposed four distinct elements of dosage for this study—1) total quantity, 2) frequency, 3) duration per use, and 4) intensity of activity during use—based on the idea that each may relate differently to mental health across individuals. The results of the study confirmed that reasoning and showed that dosage is not a single notion, and especially not one that should exclusively emphasize the total quantity of screen time. The three other sub-elements of dosage used in the research (frequency, duration, and intensity) were each important, and often to the exclusion of quantity, for individual people in the study.

Each of the five participants had different dosage profiles with respect to covariation with mental health. For Participant A (Figure 2), *duration* was the single most highly weighted dosage element, with short sessions most related to lower anxiety. For Participant B (Figure 3), who had the most complex dosage profile, three elements were roughly equally important: the *quantity* of total screens (but not the quantity of social screens), increased *duration* of sessions, and less *intensity* as measured by low switching. Each was associated with increased symptoms of depression. For Participant C (Figure 4), the *quantity* of social screens (but not the *quantity* of total screens) covaried with increased depression. For Participant D (Figure 5), lower *intensity* of involvement with the smartphone (i.e., fewer app switches), and longer session *duration* covaried with positive affect. And for Participant E (Figure 6), a lower *quantity* of total screens over the year, combined with a shorter *duration* of sessions, covaried with increased anxiety. Together, these results confirm that the *quantity* of exposure alone limits the ability to theorize about media and mental health and that other elements of dosage may provide an increase in predictive power for thinking about mental health theory as well as additional smartphone behavior that might be targeted in mental health interventions involving media.
