# Supplementary figures and images for: Person-Specific Analyses of Smartphone Use and Mental Health: Intensive Longitudinal Study
Source: JMIR Form Res. 2025 Feb 26;9:e59875. doi: 10.2196/59875 (PMC11904378; doi:10.2196/59875)

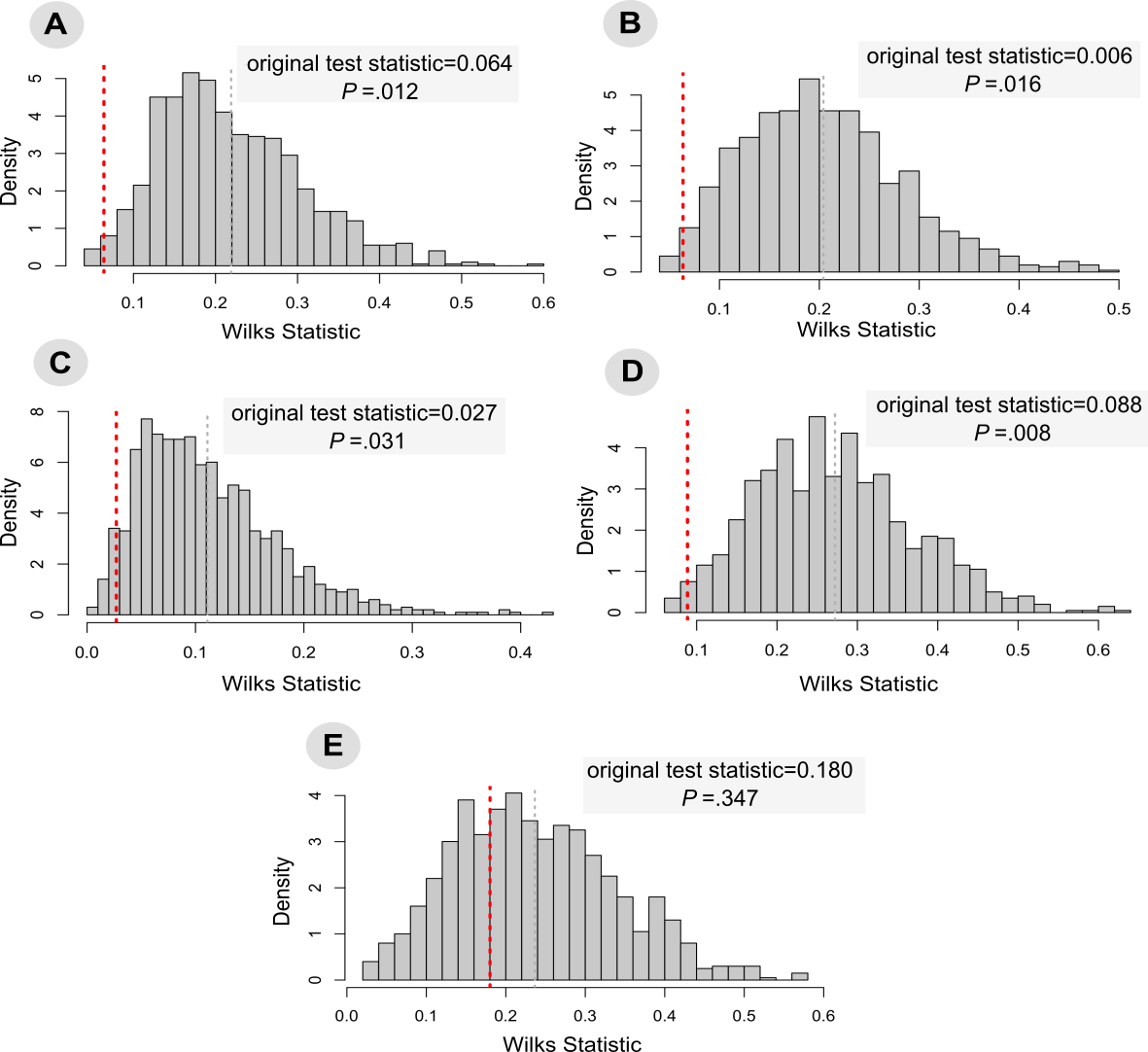

Supplement: Multimedia Appendix 2 [file formative_v9i1e59875_app2.png]
